# Anti-GM-CSF Neutralizing Autoantibodies in Colombian Patients with Disseminated Cryptococcosis

**DOI:** 10.1007/s10875-023-01451-5

**Published:** 2023-02-23

**Authors:** Carlos A. Arango-Franco, Mélanie Migaud, Isabel Cristina Ramírez-Sánchez, Karen Arango-Bustamante, Marcela Moncada-Vélez, Julián Rojas, Adrian Gervais, Santiago Patiño-Giraldo, Lizeth J. Perez-Zapata, Jesús A. Álvarez Álvarez, Julio César Orrego, Gustavo Roncancio-Villamil, Stéphanie Boisson-Dupuis, Emmanuelle Jouanguy, Laurent Abel, Jean-Laurent Casanova, Jacinta Bustamante, Andrés A. Arias, José Luis Franco, Anne Puel

**Affiliations:** 1 Laboratory of Human Genetics of Infectious Diseases, Necker Branch, Institut National de la Santé et de la Recherche Médicale (INSERM) U1163, Necker Hospital for Sick Children, 24 Boulevard du Montparnasse, Paris, France; 2 Primary Immunodeficiencies Group, Department of Microbiology and Parasitology, School of Medicine, University of Antioquia, Medellín, Colombia; 3 Imagine Institute, University of Paris Cité, Paris, France; 4 Department of Internal Medicine, School of Medicine, University of Antioquia, Medellín, Colombia; 5 Department of Internal Medicine, Division of Infectious Diseases, Pablo Tobón Uribe Hospital, Medellín, Colombia; 6 Medical and Experimental Mycology Unit, Corporation for Biological Research (CIB), Medellín, Colombia; 7 St. Giles Laboratory of Human Genetics of Infectious Diseases, Rockefeller Branch, The Rockefeller University, New York, NY USA; 8 Department of Internal Medicine, Pablo Tobón Uribe Hospital, Medellín, Colombia; 9 School of Health Sciences, Pontifical Bolivarian University, Medellín, Colombia; 10 CardioVID Clinic, Medellín, Colombia; 11 Department of Pediatrics, Necker Hospital for Sick Children, AP-HP, Paris, France; 12 Howard Hughes Medical Institute, New York, NY USA; 13 Center for the Study of Primary Immunodeficiencies, Necker Hospital for Sick Children, AP-HP, Paris, France; 14 Grupo de Inmunodeficiencias Primarias (IDPs), Facultad de Medicina, Universidad de Antioquia, Medellin, Colombia; 15 Primary Immunodeficiencies Group, Department of Pediatrics, School of Medicine, University of Antioquia, Medellin, Colombia

**Keywords:** Granulocyte–macrophage colony-stimulating factor, Pulmonary alveolar proteinosis (PAP), Meningitis, *Cryptococcus neoformans*, *Cryptococcus gattii*

## Abstract

**Background:**

Cryptococcosis is a potentially life-threatening fungal disease caused by encapsulated yeasts of the genus *Cryptococcus*, mostly *C. neoformans* or *C. gattii.* Cryptococcal meningitis is the most frequent clinical manifestation in humans. Neutralizing autoantibodies (auto-Abs) against granulocyte–macrophage colony-stimulating factor (GM-CSF) have recently been discovered in otherwise healthy adult patients with cryptococcal meningitis, mostly caused by *C. gattii*. We hypothesized that three Colombian patients with cryptococcal meningitis caused by *C. neoformans* in two of them would carry high plasma levels of neutralizing auto-Abs against GM-CSF.

**Methods:**

We reviewed medical and laboratory records, performed immunological evaluations, and tested for anti-cytokine auto-Abs three previously healthy HIV-negative adults with disseminated cryptococcosis.

**Results:**

Peripheral blood leukocyte subset levels and serum immunoglobulin concentrations were within the normal ranges. We detected high levels of neutralizing auto-Abs against GM-CSF in the plasma of all three patients.

**Conclusions:**

We report three Colombian patients with disseminated cryptococcosis associated with neutralizing auto-Abs against GM-CSF. Further studies should evaluate the genetic contribution to anti-GM-CSF autoantibody production and the role of the GM-CSF signaling pathway in the immune response to *Cryptococcus* spp.

**Supplementary Information:**

The online version contains supplementary material available at 10.1007/s10875-023-01451-5.

## Introduction

Fungi are abundant in the environment, but only a few cause human disease [1, 2]. Despite the availability of several potent antifungal drugs, the mortality associated with invasive fungal diseases (IFDs) often exceeds 50% [3]. More than 90% of all reported fungal-disease related deaths are caused by species from four genera: *Aspergillus* spp., *Candida* spp., *Pneumocystis jirovecii*, and *Cryptococcus* spp., with cryptococci accounting for almost half of all fungus-related deaths [2, 3]. IFDs occur mostly in patients with acquired immunodeficiencies, or, more rarely, in individuals with inborn errors of immunity (IEI) [1, 4, 5]. Cryptococcosis is a life-threatening IFD mostly caused by *C. neoformans* or *C. gattii*. *C. neoformans* has a worldwide distribution and is the etiological agent responsible for about 95% of all cases of cryptococcosis, in both immunocompromised and apparently immunocompetent hosts [4]. By contrast, *C. gattii* has a restricted geographic distribution and generally affects apparently healthy individuals [4]. A survey carried out in Colombia between 1997 and 2016 identified 1974 cases of cryptococcosis. Acquired immunodeficiency syndrome (AIDS) was the leading risk factor in 1505 individuals (76.2%, with an overall incidence of 1.1 cases per 1000 people with AIDS), whereas no apparent risk factor was identified in 248 cases (12.6%, overall incidence of 0.23 cases per 100,000 inhabitants) [6, 7]. The most common clinical presentation in the 1974 patients was meningitis (1600 patients, 81.1%), in both the AIDS and non-AIDS groups of patients [6].

Anti-cytokine neutralizing auto-Abs, usually found in adult patients, are considered to constitute autoimmune phenocopies of IEI [8]. Indeed, by blocking the biological function of their target cytokines, these auto-Abs cause clinical phenotypes mimicking those of inborn errors of the corresponding cytokines or their receptors [8]. Patients with neutralizing auto-Abs against interferon-gamma (IFN-γ) are mostly vulnerable to disseminated infections with non-virulent mycobacterial species and invasive non-typhoid salmonellosis [8]. Their clinical phenotype therefore resembles that of patients presenting with Mendelian susceptibility to mycobacterial diseases (MSMD) due to mutations of 20 genes controlling the production of, or the response to, IFN-γ [9, 10]. IL-6 neutralizing auto-Abs have been described in patients suffering from severe cutaneous and invasive staphylococcal and pneumococcal diseases [11]. These clinical phenotypes resemble that of patients with IEI impairing the response to or the production of IL-6 (e.g., autosomal recessive (AR) IL-6R, autosomal dominant (AD) IL6ST/gp130, AD STAT3, AR ZNF341, AR MYD88, or AR IRAK4 deficiencies) [12]. Auto-Abs against IL-17A and IL-17F (and IL-22) have been reported to underlie chronic mucocutaneous candidiasis (CMC) in patients with autoimmune polyendocrine syndrome type 1 (APS-1), paving the way for the identification of inborn errors of IL-17 immunity in patients with isolated or syndromic CMC (e.g., AD IL-17F, AR IL-17RA, AR IL-17RC, AR ACT1, AD MAPK8, AR IL-23R, AR c-Rel, and AR ZNF341 deficiencies, or AD STAT1 gain-of-function [GOF]) [13–15]. Auto-Abs against type I IFNs were first identified in the early 1980s in patients treated with IFN-α or IFN-β, with systemic lupus erythematosus, with thymic abnormalities (e.g., thymoma), or with various IEIs (e.g., APS-1 and AR AIRE deficiency) [8]. These auto-Abs were long thought to be clinically silent, but were recently shown to underlie critical COVID-19 pneumonia [16], adverse reactions to yellow fever live-attenuated vaccine [17], critical influenza pneumonia [18], or severe varicella-zoster virus diseases [19]. The associated clinical phenotype resembles that of patients with IEI of type I IFN underlying severe viral diseases [8, 20].

Anti-GM-CSF auto-Abs cause pulmonary alveolar proteinosis (PAP), a severe lung disease characterized by the accumulation of surfactant in the alveoli, with progressive respiratory failure and an increase in the risk of infection [21], probably due to an impairment of the terminal differentiation of alveolar macrophages affecting their ability to catabolize surfactant, and to protect the host against infectious diseases [21]. Patients with acquired PAP are also prone to recurrent common pulmonary infections, possibly secondary to the underlying lung dysfunction. They are also vulnerable to infections caused by intracellular pathogens, including *Mycobacterium* spp*.* complex, *Nocardia* spp.,* Histoplasma capsulatum*, and *Cryptococcus* spp. [22]. In addition, auto-Abs against GM-CSF have been reported in adult patients with disseminated diseases mostly due to *Nocardia* spp. or *C. gattii* with or without PAP manifestations [23–27]. In this context, we investigated three previously healthy HIV-negative Colombian adults with cryptococcal meningitis. One of these patients presented PAP 9 months later, followed 5 years later by pulmonary tuberculosis.

## Materials and Methods

### Subjects

This study was conducted according to the “Scientific Standards for Technical and Administrative Health Research” established in the Colombian Ministry of Health Resolution 008430 of 1993 and approved by the local review board of the Universidad de Antioquia (F8790-07–0010) and Necker Hospital for Sick Children, France. All patients or their family members provided written informed consent.

### Detection of Anti-GM-CSF Auto-Abs by ELISA

Briefly, 96-well plates (Nunc Maxisorp, Thermo Fisher Scientific) were coated by incubation overnight at 4°C with 1 µg/mL rhGM-CSF or rhIFN-γ (R&D Systems). The plates were washed in PBS 0.005% Tween, blocked with 1 × PBS supplemented with 5% nonfat milk powder, washed, and incubated for 2 h at room temperature with 1:50, 1:250, and 1:1,000 dilutions of plasma from patients and healthy controls, or plasma from a patient with cryptococcal meningitis and a PAP patient previously shown to have high titers of anti-GM-CSF auto-Abs, as a positive control. The plates were washed and horseradish peroxidase (HRP)–conjugated Fc-specific IgG (polyclonal goat antiserum against human IgG, Nordic Immunological Laboratories) was added to a final concentration of 1 µg/mL. Plates were incubated for 1 h at room temperature and washed. Substrate was added, and optical density was measured. The antibody specificity controls were plasma samples from a patient with high titers of auto-Ab against IFN-γ, and a patient with APS-1 and high titers of auto-Ab against IL-17A, IL-17F, IL-22, IFN-α, and IFN-ω.

### Plasma Inhibition of GM-CSF-induced STAT5 Phosphorylation

Human peripheral blood mononuclear cells (PBMCs) from healthy controls were isolated from whole blood by Ficoll-Hypaque density centrifugation (Amersham-Pharmacia-Biotech, Sweden). The cells were counted and plated at 2 × 10^6^ cells/well in 96-well V-bottom plates (Thermo-Fisher-Scientific), in 100 µL RPMI (GibcoBRL, Invitrogen), supplemented with 10% fetal bovine serum (FBS) (GibcoBRL, Invitrogen) or 100 µL RPMI supplemented with 10% plasma from patients or controls. PBMCs were left unstimulated or were stimulated with 5 to 80 ng/mL rhGM-CSF or 100 ng/mL rhIL-3 (Miltenyi-Biotec) for 30 min at 37°C, and the cells were then fixed permeabilized with a fixation/permeabilization kit (eBioscience). Extracellular labeling was performed with CD14-Pacific Blue and CD4-FITC (Sony-Biotechnology, clones M5E2 and RPA-T4, respectively). Cell viability was determined with the Aqua Dead Cell Stain Kit (Thermo-Fisher-Scientific), and STAT5 phosphorylation (p-STAT5 levels) was assessed by intracellular staining with Phospho-Flow PE Mouse Anti-p-STAT5 (pY694) antibody (BD-Biosciences). Data were collected with a Gallios flow cytometer (Beckman-Coulter) and analyzed with FlowJo software v.10.6.2 (Becton–Dickinson).

### Neutralization Activity of Type I IFNs

The plasma blocking activity against type I IFNs (13 IFN-α subtypes, IFN-β, and IFN-ω) was determined with a luciferase reporter assay. Briefly, HEK293T cells, cultured in DMEM ﻿(Thermo Fisher Scientific) with 10% FBS were transfected in the presence of X-tremeGene9 transfection reagent (Sigma-Aldrich) for 24 h with a human ISRE-luciferase plasmid in the pGL4.45 backbone and a plasmid constitutively expressing *Renilla* luciferase for normalization (pRL-SV40). Then, cells were left unstimulated or were stimulated with IFNs (IFN-α subtypes (Miltenyi Biotec), IFN-ω (Peprotech), or IFN-β (Peprotech)) at 100 pg/mL (for IFN-α or IFN-ω) or at 1ng/mL (for IFN-β) for 16 h at 37°C in the presence of 10% of healthy control or patient plasma diluted in DMEM with 2% FBS. Luciferase activity was then assessed in the Dual-Luciferase® Reporter 1000 assay system, according to the manufacturer’s protocol (Promega). Raw luciferase induction was calculated as firefly luciferase activity normalized against *Renilla* luciferase activity, and this raw luciferase induction was normalized against the non-stimulated luciferase induction.

## Results

### Case Reports

**Fig. 1:**
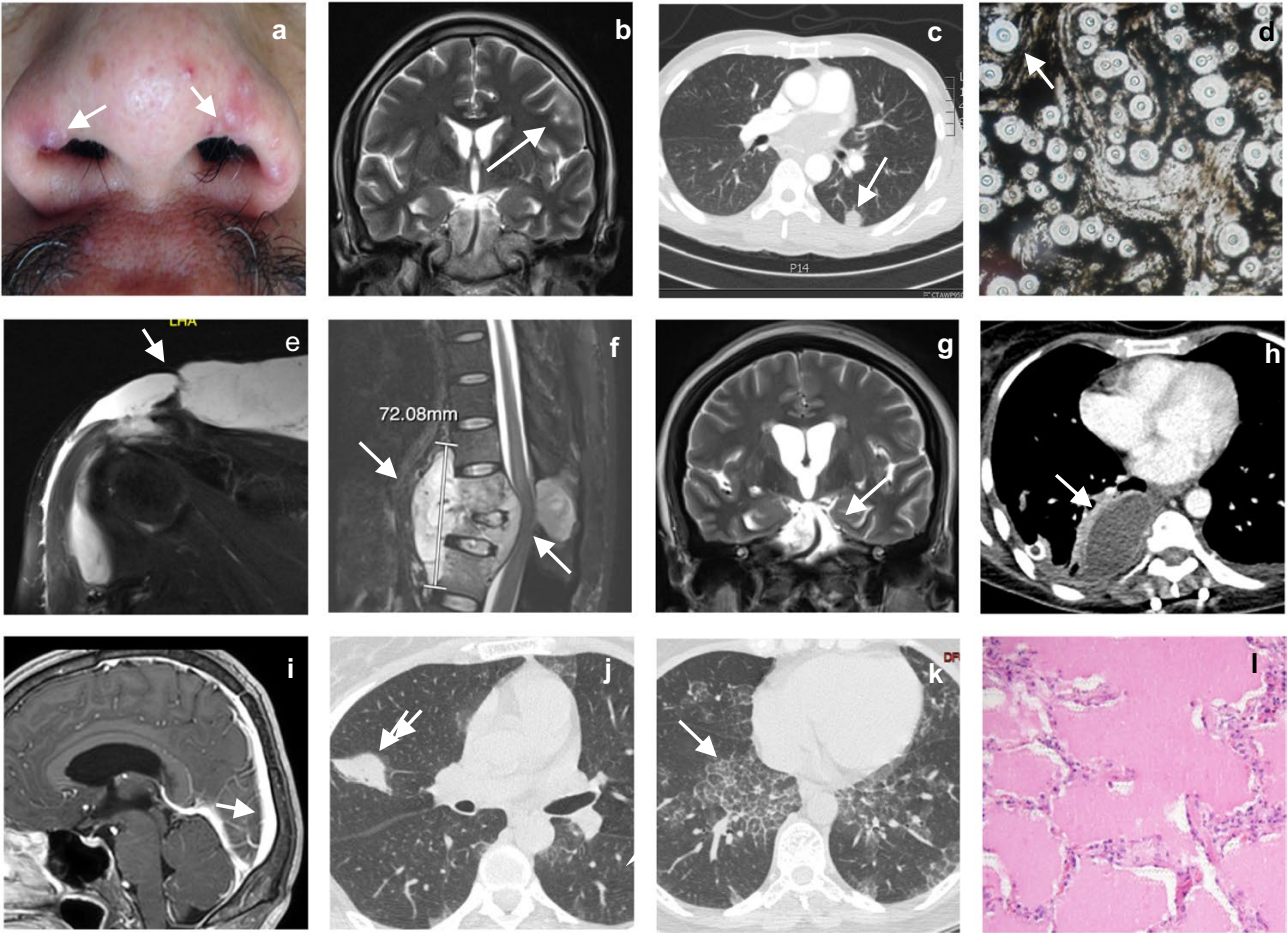
Clinical, microbiologic, and radiologic findings and tissue staining results for patients. Patient 1: **a** Papular skin lesions on the nasal ala (arrows); **b** Brain CE-MRI showing multiple nodular lesions affecting the supratentorial areas and the basal ganglia (arrows); **c** Chest CE-CT scan showing a mediastinal mass surrounding vessels (arrow) and a pulmonary nodule in the upper part of the left lower lobe (arrow); the fistula is not shown; **d** India ink staining of the mediastinal mass biopsy specimen, showing unstained thick mucopolysaccharide capsules (arrow) from *Cryptococcus* (40X). Patient 2: **e** Whole-body CE-MRI: coronal T2-weighted sequence showing hyperintense right supraclavicular fluid collection in the right shoulder affecting the acromioclavicular joint, with infiltration of the bursa and bone (arrows); **f** Sagittal T2-weighted sequence showing fluid collection extending to the retroperitoneal, dural, and posterior mediastinum, together with spinal cord compression and instability and bone destruction between T9 and T11, with 90% collapse of the space between T10 and T11 (arrows); **g** Brain CE-MRI showing acute hydrocephalus and basal meningitis with cranial nerve involvement (arrow); **h** Chest CT-scan showing a mass protruding into the right mediastinal space and bilateral mild pleural effusion (arrow). Patient 3. **i** Brain MRI sagittal T2-plane showing pathological meningeal enhancement and vasogenic edema (arrow); **j** Chest CE-CT scan showing an irregular pulmonary nodule in the upper right lobe and a diffuse ground-glass pattern (arrow); **k** Chest CE-CT scan showing patchy bilateral ground-glass opacities with thickening of the interlobular septa with a “crazy-paving” pattern, predominantly in the lower zone (arrow); **l** Abundant extracellular proteinaceous periodic acid-Schiff (PAS)-positive material on lung biopsy (100 ×)

Patient 1 (P1) is an otherwise healthy 41-year-old man from Colombia (South America) who presented at the age of 34 years with progressive precordial pain with dysphagia, dry cough, moderate dyspnea, and a weight loss of 26 kg. After a convulsive episode, he was admitted to the emergency room, and physical examination revealed neck stiffness, a grade 2 systolic pulmonary murmur, and purple skin papules on his nose (Fig. 1a). Lumbar puncture revealed intracranial hypertension (ICH) and cerebrospinal fluid (CSF) abnormalities; India ink staining of the CSF and a serum cryptococcal antigen lateral flow assay (CrAg-LFA) were positive for *Cryptococcus* spp. (Table 1). Contrast-enhanced magnetic resonance imaging (CE-MRI) of the brain revealed multiple nodular lesions in the supratentorial areas and the basal ganglia that were consistent with cryptococcosis (Fig. 1b). Gram and India ink staining of sputum samples revealed the presence of encapsulated yeasts consistent with *Cryptococcus* spp. (Table 1). Contrast-enhanced computed tomography (CE-CT) of the chest revealed a peripheral nodule in the upper segment of the lower lobe of the left lung and a mediastinal mass (Fig. 1c). A biopsy of the mediastinal mass was performed by video-assisted thoracoscopic surgery (VATS), and the specimen was stained with India ink, revealing the presence of encapsulated yeasts consistent with *Cryptococcus* spp. (Fig. 1d). An echocardiogram revealed a mobile mass within the left atrium suggestive of mycotic endocarditis (not shown). Blood tests revealed leukocytosis with neutrophilia and high C-reactive protein (CRP) levels. An HIV test was negative, and flow cytometry analyses of lymphocyte subpopulations and serum immunoglobulins (Ig) were normal, with the exception of high IgE levels (Tables 1 and 2). An upper gastrointestinal (GI) tract endoscopy detected an esophageal ulcer positive for *Cryptococcus* spp., and cultures of CSF and the mediastinal mass on Sabouraud dextrose agar (SDA) grew *C. neoformans* (Tables 1 and 2), confirming the diagnosis of disseminated cryptococcosis. The patient was treated with a 6-week course of IV liposomal amphotericin B (LAmB) (250 mg/day), and 5 flucytosine (5-FC) (1 g qid), according to susceptibility test results, and esophageal and bronchial stents were implanted to close the fistula. However, serial lumbar punctures revealed persistent ICH after 4 weeks, leading to the insertion of a ventriculoperitoneal shunt (VPS). Finally, negative cultures of CSF, blood, and bronchial and esophageal tissues were obtained, and negative results were obtained for cytological analyses of bronchoalveolar lavage (BAL), ruling out a diagnosis of PAP. A clinical improvement was observed, and the patient was discharged on oral FLC (800 mg/day), on which he remains, and is doing well.

**Table 1:** Blood and CSF laboratory parameters and microbiologic and pathology analyses of various tissues from the three patients at diagnosis

	Patient 1	Patient 2	Patient 3
Age at onset (years), Sex	34, male	40, female	44, female
Hemogram			
Hemoglobin (g/dL)	13 (ref. 13–17)	14 (ref. 12–15)	**15** (ref. 12–15)
Hematocrit (%)	45.1 (ref. 40–52)	41 (ref. 36–47)	44 (ref. 36–47)
WBC (ref. 4–10 × 10^9^/L)	**13.7**	9.2	**15.1**
Neutrophils (ref. 2–8 × 10^9^/L)	**8,8**	**4.8**	**11.3**
Lymphocytes (ref. 1–4 × 10^9^/L)	1.2	**0.9**	2.6
Monocytes (ref. 0.2–0.8 × 10^9^/L)	0.6	0.6	0.6
Eosinophils (ref. < 0.5 × 10^9^/L)	0.0	0.1	**1.3**
Platelets (ref. 150–450 × 10^9^/L)	428	328	**506**
Blood urea nitrogen (ref. 8–21 mg/dL)	14	13	13
Creatinine (ref. 0.8–1.3 mg/dL)	0.95	0.71	0.75
C-reactive protein (ref. 0.01–0.82 mg/dL)	**3.7**	**5.0**	0.7
HIV serum antibodies (ELISA)	Negative	Negative	Negative
HIV serum viral load (HIV-1 RNA/mL)	Negative	Negative	Negative
Cerebrospinal fluid (CSF)			
Total protein (ref. 5–45 mg/dL)	**82**	**242**	**68**
Glucose (ref. 45–80 mg/dL)	**22**	**31**	48
WBC (ref. 0–5 cells/µL)	**200**	**50**	**92**
Opening pressure (ref. 10–25 cm H_2_O)	**47**	20	21
ADA (15–60 mg/dL)	**13**	24	**3**
Microbiologic and histologic tests			
Gram/India ink staining of selected samples	Positive for *Cryptococcus spp*. (sputum, CSF)	Positive for *Cryptococcus spp*. (CSF)	Positive for *Cryptococcus spp*. (CSF)
Serum cryptococcal antigen test and titer	1:512 (LFA)	1:256	1:128
Sabouraud dextrose agar cultures of selected samples and biopsies	All positive for *C. neoformans* (sputum, CSF, peripheral blood, BAL, mediastinal mass, esophageal ulcer, papular skin lesions, soft tissues and bone)	Positive for *C. neoformans* (CSF and mediastinal mass)	Positive for *C. gattii* (CSF and mediastinal mass)
Urea hydrolysis and L-canavanine glycine bromothymol blue (CGB)	Positive for *C. neoformans* (CSF and mediastinal mass)	Positive for *C. neoformans* (CSF and mediastinal mass)	Positive for *C. gattii* (CSF and mediastinal mass)
Minimum inhibitory concentration (MIC)	FLC < 2	FLC < 2	FLC 16 VRC < 0.125
Pathology findings and silver methenamine and mucicarmine staining of selected tissues	Severe active chronic inflammation Silver methenamine and mucicarmine staining positive for blastoconidia (esophagus, skin and mediastinal mass)	Extensive coagulation necrosis, chronic inflammation Silver methenamine and mucicarmine staining positive for blastoconidia (mediastinal mass)	Histiocytic pneumonia, chronic inflammation Silver methenamine and mucicarmine staining positive for blastoconidia (lung mass)
Other			
Aerobic cultures	Negative	Negative	Negative
PCR mycobacteria/*Mycobacterium* cultures	Negative	Negative	Negative at diagnosis Positive for *M. tuberculosis* 5 years later

Values in bold are outside the normal range for age

*FLC* fluconazole, *VRC* voriconazole, *BAL* bronchoalveolar lavage, *LFA* lateral flow assay, *CSF* cerebrospinal fluid

**Table 2:** Flow cytometry of lymphocyte subsets in peripheral blood, serum immunoglobulins, and specific antibodies

	Patient 1*	Patient 2	Patient 3*	Ref. values for age**
WBC (cells/µL)	**7,645**	NR	6,270	5,900 (4,600–7,100)
Lymphocytes (%)	**20**	NR	30.2	32 (28–39)
Lymphocytes (cells/ µ L)	1,529	NR	1,894	2,300 (1,200–4,100)
Lymphocyte subsets				
CD3^+^ (%)	56	NR	71.7	67 (50–91)
CD3^+^(cells/µL)	856	876 (700–2,100)	1,358	1,500 (780–3,000)
CD3^+^/CD4^+^ (%)	36.2	NR	44.3	42 (28–64)
CD3^+^/CD4^+^ (cells/µL)	553	613 (300–1,400)	839	1,000 (500–2,000)
CD3^+^/CD8^+^ (%)	18.7	NR	23.5	22 (12–40)
CD3^+^/CD8^+^ (cells/µL)	286	265 (200–900)	445	500 (200–1,200)
CD3^+^/CD4^+^/CD8^+^ (%)	0.4	NR	0.4	0.26 (0.08–0.94)
CD3^+^/CD4^+^/CD8^+^ (cells/µL)	4	NR	6	12 (2–60)
CD4/CD8 ratio	1.9	2.3	1.9	1.9 (1.0–3.6)
CD19^+^ (%)	13.4	NR	8.5	10 (4–28)
CD19^+^ (cells/µL)	205	NR	161	230 (64–820)
CD3^−^CD16^+^/CD56^+^ (%)	24.1	NR	16.5	15 (5–49)
CD3-CD16^+^/CD56^+^	368	NR	312	340 (100–1,200)
CD45^+^/CD14^+^ (%)	7.7	NR	5.7	3–8
CD45^+^/CD14^+^ /µL	591	NR	359	100–8,000
Serum immunoglobulins				
IgG (mg/dL)	1467 (540–1,822)	1098	ND	(814–2,047)
IgA (mg/dL)	200 (63–484)	88	ND	(81–538)
IgM (mg/dL)	213 (22–240)	154	ND	(42–600)
IgE (IU/mL)	**1,151** (0–100)	ND	ND	
HB IgG (mU/mL)	0.08 (< 10)	ND	ND	
Rubella IgG (mU/mL)	187 (< 10)	ND	ND	

Values in bold are outside the normal ranges for age

*ND* not done, *NR* not reported

*Flow cytometry of lymphocyte populations performed on peripheral blood lymphocytes (PBLs) stained with fluorochrome-labeled mAbs. Cells were collected on a FACS Canto II (BD Biosciences, San José, CA), and the data were analyzed with FlowJo v8.2 (TreeStar, Ashland, OR) by gating on CD45^+^ leukocytes. Reference values for lymphocyte subsets from Schatorjé EJH, et al. *Scand. J Immunol*. 2012 vol. 75 (4) pp. 436–44

**For patient 2, only results from the BD Tritest™ (BD Biosciences) were available and reference values are as indicated in the hospital records

Patient 2 (P2) is a 46-year-old otherwise healthy woman from Colombia (South America) who presented at the age of 40-year-old shoulder pain of four months’ duration radiating to the lower back, together with paresthesia of the lower abdomen and difficulty walking. Physical examination revealed a painful abdomen with bilateral positive Lasègue sign, sensory and motor deficit, a decrease in the muscle strength of both lower limbs, right Achillean reflex clonus, and bilateral positive Babinski sign. The patient suffered from permanent bilateral vision loss of unknown cause that had started 4 years previously, with lens opacification in the left eye and a corneal leukoma in the right eye. CE-MRI revealed extensive fluid collections in the right shoulder and lumbar spine, extending to the retroperitoneal, dural, and posterior mediastinum, along with spinal cord compression and instability and bone destruction between T9 and T11 (Fig. 1e, f). P2 underwent intraoperative lavage and debridement with T9-T11 fixation, and the tissue samples excised were stained with silver methenamine and mucicarmine, indicating the presence of *Cryptococcus* spp.; *C. neoformans* grew in cultures of CSF and the mediastinal mass on SDA (Table 1). Brain CE-MRI showed hydrocephalus and acute meningitis (Fig. 1g), and CSF analysis after lumbar puncture revealed high total protein concentration, normal glucose concentration, high levels of leukocytes, and the CrAg assay was positive for *Cryptococcus spp.* (Table 1). Initial blood testing revealed a normal whole blood count (WBC), mild lymphopenia, and high CRP concentration; an HIV test was negative (Table 1). T-lymphocyte counts, CD4^+^/CD8^+^ T-cell ratio, and serum Ig levels were normal (Table 2). P2 was treated with IV deoxycholate AmB (DAmB) (42 mg/day) and oral 5-FC (1,500 mg qid) for 14 days, followed by oral FLC (800 mg/day) for 12 weeks, and was discharged on oral FLC (200 mg/day) plus analgesics. Seven months later, the patient was re-hospitalized due to severe back pain radiating to the left hypochondrium. The chest CT scan revealed a mediastinal mass and pleural effusion (Fig. 1h), and P2 underwent VATS for lung pleurectomy, decortication, and biopsy of the mediastinal mass. Silver methenamine and mucicarmine staining of the mediastinal mass revealed *Cryptococcus* spp. The patient was placed on IV DAmB (42 mg/day) for 2 weeks but developed respiratory failure due to loculated pleural effusion of the right hemithorax with secondary lung collapse, requiring a new pleurectomy with decortication. Throughout P2’s disease, radiological and BAL studies have consistently ruled out pulmonary alveolar proteinosis (not shown). The patient remains free of signs and symptoms of infection and is not currently receiving antifungal agents; she is, however, permanently paraplegic.

Patient 3 (P3) is a 44-year-old previously healthy woman from Colombia who developed progressive whole-head headaches with photophobia, nausea, and vomiting in 2014. Physical examination was unremarkable except for neck stiffness with no neurologic focalization. Brain CE-MRI findings were consistent with meningitis (Fig. 1i), and lumbar puncture revealed a normal opening pressure and a CSF with a mild increase in protein levels, normal glucose concentration, and a high level of leukocytes (Table 1). A non-contrast chest CT-scan revealed a right lung mass in contact with the pleura and a diffuse bilateral ground-glass pattern (Fig. 1j). VATS was performed to obtain samples of the lung mass and the affected pleura. Silver methenamine staining of the lung sample was consistent with the presence of *Cryptococcus* spp., and *C. gattii* grew in cultures of lung and pleura tissues on SDA, confirming the diagnosis of disseminated cryptococcosis (Table 1). Initial blood tests revealed moderate leukocytosis with neutrophilia and eosinophilia, thrombocytosis, and normal CRP levels; an HIV test was negative (Table 1). Flow cytometry analysis showed the proportions of the principal lymphocyte subpopulations to be within the range of normal values, and serum Ig levels were not evaluated (Table 2). The patient started a 6-week course of treatment with IV DAmB (1 mg/kg/day) plus oral FLC (600 mg/day). However, antimicrobial susceptibility testing for FLC revealed a minimal inhibitory concentration (MIC) of 16 µg/mL (Table 1). P3 was therefore transferred onto suppressive therapy with voriconazole (VRC) at a dose of 200 mg/12 h for 9 months, leading to the resolution of meningitis. A few months later, she developed a progressive dry cough and severe dyspnea and lost 7 kg in body weight. A new lumbar puncture and hemogram yielded values within the normal range. However, a CrAg assay on CSF was positive for *Cryptococcus* spp. High-resolution CT (HRCT) of the lungs revealed an increase in interstitial involvement, with a “crazy-paving” pattern (Fig. 1k). A right lung biopsy was therefore performed by VATS, and histological analysis demonstrated the presence of abundant foamy histiocytes with myxoid material and small oval transparent structures consistent with blastoconidia (not shown). In addition, the alveolar spaces were occupied by amorphous eosinophilic material composed of histiocytes, cholesterol crystals, and a proteinaceous material positively stained with periodic acid-Schiff (PAS) staining, consistent with PAP (Fig. 1l). Ziehl–Neelsen (ZN) staining for mycobacteria was negative, and lung tissue cultures were negative for aerobic bacteria, fungi, and mycobacteria. The patient received suppressive therapy with oral posaconazole (POS; 300 mg/day) for 3 years and remained clinically stable. Five years later, she consulted again for a persistent dry cough and weight loss. Chest HRCT showed a progression of pulmonary damage, with ground-glass and bilateral multilobar consolidations, solid nodules of up to 8 mm in diameter, and centrilobular nodules with a “budding tree” morphology. BAL and lumbar puncture were normal, and cultures were negative. VATS was performed on the right lower lobe, and histological analysis of the specimen revealed the presence of multiple giant epithelioid cells with caseous necrosis. Methenamine silver and ZN staining was negative, but PCR (GeneXpert, Cepheid®) and cultures for mycobacteria were positive for multisusceptible *Mycobacterium tuberculosis*. P3 received a directly observed treatment short (DOTS) regimen with rifampicin (R), isoniazid (H), pyrazinamide (Z), and ethambutol (E) for 2 months, followed by 4 months of R-H treatment with clinical improvement, but with dyspnea on exertion. P3 has not undergone whole-lung lavage or required oxygen supplementation, and she remains stable.

### Detection of High Titers of Neutralizing Auto-Abs against GM-CSF in Patients’ Plasma

**Fig. 2:**
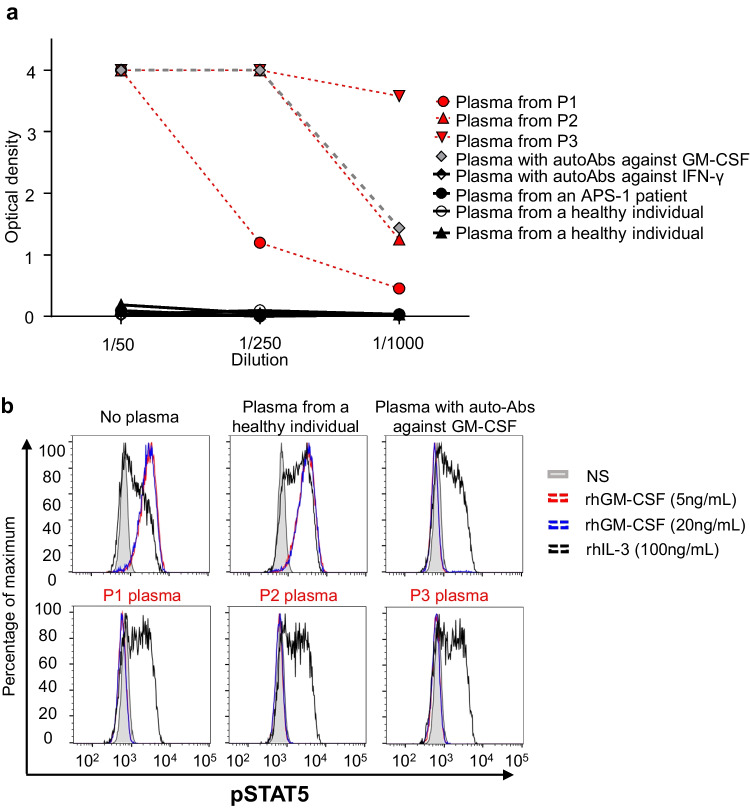
Anti-GM-CSF neutralizing auto-Abs. **a** Anti-GM-CSF auto-Ab titers in plasma from the three patients diluted 1:50, 1:250, and 1:1,000 (red), healthy individuals (black) and two patients previously shown to have anti-GM-CSF (grey) or anti-IFN-γ (black and white) auto-Abs, and an APS-1 patient with auto-Abs against IL-17A, IL-17F, IL-22 IFN-α, and IFN-ω (black). **b** STAT5 phosphorylation (p-STAT5), assessed by flow cytometry, upon the stimulation with GM-CSF or IL-3 of control PBMCs, in the absence of plasma, or in the presence of a 1:10 dilution of plasma from a healthy individual, or from the patients. NS non-stimulated

The etiological nature and severity of the cryptococcal infections in these three previously healthy adult patients prompted us to evaluate the possibility that neutralizing anti-GM-CSF auto-Abs might underlie the infectious diseases observed in these patients. We performed an ELISA for the detection of these auto-Abs in plasma samples from these three patients. As a control, we used plasma from a patient with PAP and high titers of neutralizing anti-GM-CSF auto-Abs (positive control). We also included plasma from a patient with high titers of anti-IFN-γ neutralizing auto-Abs, an APS-1 patient with high titers of anti-IL-17A, IL-17F, and IL-22 auto-Abs and two healthy individuals. All three patients had high levels of anti-GM-CSF auto-Abs (with P3 plasma showing the highest levels), in the range of the positive control, whereas none of the healthy individuals, the APS-1 patient, or the patient with auto-Abs against IFN-γ had auto-Abs against GM-CSF (Fig. 2a and Supplementary Fig. 1). We detected no neutralizing auto-Abs against type I IFNs in the three patients in the testing conditions used (Supplementary Fig. 2). We then assessed the neutralizing activity of the plasma samples from the three patients by stimulating PBMCs from healthy individuals ex vivo with rhGM-CSF or IL-3 as a positive control to induce GM-CSF receptor-mediated or IL-3R-mediated phosphorylation of STAT5 (pSTAT5) in the presence of 10% plasma from healthy individuals, or from the patients, evaluated by flow cytometry. Unlike plasma from healthy individuals, plasma from the three patients, incubated with increasing concentrations (5 - 80 ng/mL) of GM-CSF prevented GM-CSF-induced STAT5 phosphorylation, whereas the level of IL-3-induced STAT5 phosphorylation was similar in cells incubated with controls’ or patients’ plasma (Fig. 2b, Supplementary Fig. 3). Last, we assessed the neutralizing capacities of the patients’ plasmas, diluted 1:10, 1:100, 1:1,000, in the presence of 5ng/mL GM-CSF. P3’s plasma exhibited the highest neutralizing capacity, able to block GM-CSF-induced STAT5 phosphorylation when diluted 1:100, whereas P1’s and P2’s plasmas were either no more neutralizing or partially neutralizing at the 1:100 dilution, respectively. These results suggested a correlation between the auto-Ab titers, as assessed by ELISA, and their neutralizing capacity (Supplementary Fig. 4). Altogether, these results strongly suggest that the presence of circulating neutralizing auto-Abs against GM-CSF was responsible for the disseminated cryptococcosis of these patients.

## Discussion

Human GM-CSF is produced by alveolar epithelial cells, monocytes/macrophages, activated T cells, B cells, NK cells, endothelial, epithelial, and fibroblast cells and promotes the development of bone marrow-derived macrophages and granulocytes [28, 29]. Alveolar macrophages (AM) serve as the first line of defense against inhaled microbial pathogens and toxins, removing inhaled debris, excess surfactant, and apoptotic cells [21]. GM-CSF promotes the differentiation and function of pulmonary AM, as shown by knockout mice, which develop PAP and AM abnormalities, including defects of migration, phagocytosis, microbicidal activity [30], and impaired recruitment and activation of other inflammatory cells, and decreased oxygen radical production [31]. A GM-CSF knockout mouse model with progressive cryptococcal lung infection displayed low levels of leukocyte recruited and of Th2 and Th17 responses in the lung, and low total numbers, activation, and localization of dendritic cells and macrophages to the microanatomic sites of alveolar infection [32]. Collectively, these data suggest that GM-CSF is crucial for the local differentiation, accumulation, activation, and alveolar localization of lung dendritic cells and AM in mice with cryptococcal lung infection.

The three patients investigated here were otherwise healthy adults who developed disseminated cryptococcosis in association with high titers of anti-GM-CSF neutralizing auto-Abs. Anti-GM-CSF neutralizing auto-Abs block GM-CSF receptor signaling, thereby affecting the terminal differentiation of AM in the lungs and their ability to catabolize surfactant and to perform their host defense functions [33]. Anti-GM-CSF neutralizing auto-Abs are more frequently reported in adult patients infected with *C. gattii* than in those infected with *C. neoformans* [23–25]. Interestingly, two of the three patients described here developed adult-onset disseminated disease due to *C. neoformans*. One of the three patients (P3) was diagnosed with PAP nine months after developing disseminated cryptococcosis due to *C. gattii*. PAP is characterized by an accumulation of alveolar surfactant, resulting in respiratory impairment, an increase in the risk of pulmonary fibrosis, and opportunistic infections [21]. Early-onset PAP (inherited PAP) results from inborn errors of immunity due to biallelic mutations of genes that disrupt GM-CSF signaling [12], or from inborn errors of surfactant metabolism due to mutations of genes involved in surfactant production and function in alveolar epithelial cells [21]. By contrast, autoimmune PAP has an essentially adult onset, with more than 90% of cases due to high serum levels of neutralizing anti-GM-CSF auto-Abs [34], whereas about 5 to 10% of the remaining adult PAP cases may be secondary to underlying conditions, such as hematological disorders, cancers, chronic inflammatory syndrome, and chronic infections [21]. In adults, PAP may precede or follow infections, such as pulmonary or disseminated cryptococcosis with lung involvement [35]. Lung and disseminated infections with various fungi, including *Cryptococcus* spp., *Nocardia* spp., and *Aspergillus* spp., may therefore be an early sign of a risk of developing PAP in the future [22, 25, 36].

Only one adult patient with disseminated cryptococcosis and high serum levels of anti-GM-CSF auto-Abs has, to our knowledge, developed pulmonary tuberculosis within 1 year of the diagnosis of cryptococcosis [25]. P3 was diagnosed with pulmonary tuberculosis 6 years after the diagnosis of cryptococcosis and 5 years after the initial diagnosis of autoimmune PAP. Interestingly, P3, who displayed the most severe disease, presented with the highest titers and neutralizing activity of anti-GM-CSF auto-Abs. Immune responses to mycobacteria are controlled principally by IFN-γ, as demonstrated by findings for patients carrying mutations of any of the 20 genes leading to MSMD [9, 10]. In a single-cell model of *M. tuberculosis* killing by primary human monocyte-derived macrophages, Brison et al. recently demonstrated that GM-CSF enhances the control of *M. tuberculosis*, as GM-CSF blockade rendered macrophages more permissive to *M. tuberculosis* growth, whereas the addition of GM-CSF increased bacterial control [37]. Furthermore, GM-CSF-deficient mice are highly susceptible to infection by *M. tuberculosis* [38, 39]. Anti-GM-CSF neutralizing auto-Abs may, therefore, also potentially increase susceptibility to *M. tuberculosis* infection. In addition, GM-CSF promotes human macrophage differentiation in vitro and fine tunes macrophage inflammatory state, enhancing mycobacterial control by activating antimicrobial pathways and amplifying an inflammatory environment mediated by IL-1β [37, 40]. Therefore, it is possible that, in addition to the PAP, frequently associated with structural lung disease, the neutralizing auto-Abs against GM-CSF have participated to P3’s susceptibility to M. tuberculosis infection. In PAP, the abolition of AM function affects the clearance of surfactant, leading to a structural lung disease that may increase susceptibility to *M. tuberculosis*. The endemic distribution of both pathogens (*C. neoformans* and *M. tuberculosis*) in Colombia may render patients with anti-GM-CSF neutralizing auto-Abs more susceptible to these diseases. Recent evidence suggests that additional host genetic factors, such as HLA haplotype, may result in the development of anti-cytokine auto-Abs and susceptibility to infections in the context of adult-onset autoimmune-induced immunodeficiency [41]. Sakaue et al. have shown that there may be a genetic contribution to the pathogenesis of autoimmune PAP. They conducted a genome-wide association study in patients of Japanese ancestry and demonstrated an association of the HLA-DRB1*08:03 allele with a risk of autoimmune PAP and high levels of anti-GM-CSF auto-Abs [42]. However, a study of 47 European patients with PAP found that no HLA allele was associated with these auto-Abs [43]. Pulmonary and disseminated cryptococcosis in previously healthy HIV-negative adults, with or without PAP, should lead to investigations of a possible autoimmune etiology.

## Supplementary Information

Below is the link to the electronic supplementary material.

Supplementary file1 (PPTX 922 KB) Supplemental figure 1: Anti-GM-CSF auto-Ab titers in plasmas, diluted 1/50, from the three patients (P1-P3), healthy European individuals (n=35, white circles), patients previously shown to have anti-GM-CSF (n=5, gray diamond) or anti-IFN-γ (n=1, black and white diamond) auto-Abs, an APS-1 patient with auto-Abs against IL-17A, IL-17F, IL-22, IFN-α, and IFN-ω (n=1, black circle). Supplemental figure 2: Absence of neutralizing auto-Abs against type I IFNs in the plasma of the patients. Relative luciferase activity (RLA) ratio after stimulation with each of the IFN-α subtypes or IFN-ω at a concentration of 100 pg/mL, with a 1:10 dilution of plasma from seven healthy controls (negative control), an APS-1 patient (positive control), and the three patients with neutralizing anti-GM-CSF auto-Abs. Results are normalized against the RLA value obtained in the presence of plasma but absence of stimulation. Supplemental figure 3: Plasma from the three patients diluted 1:10 neutralizes up to 80 ng/mL GM-CSF. pSTAT5 levels assessed by flow cytometry on total PBMCs from a healthy donor stimulated with 5 ng/mL, 20 ng/mL, 40 ng/mL, or 80 ng/mL of GM-CSF or 100 ng/mL of IL-3, in the absence of plasma, or in the presence of a 1:10 dilution of plasma from two healthy controls (negative control), a patient with alveolar proteinosis PAP (positive control), or one of the three patients. NS: non-stimulated. Supplemental figure 4: STAT5 phosphorylation (p-STAT5), assessed by flow cytometry, upon stimulation with 5ng/mL rhGM-CSF (red) or 100ng/mL rhIL-3 (black) of control PBMCs in the presence of a 1:10, 1:100 or 1:1,000 dilution of plasma from two healthy individuals, three individuals previously described with neutralizing autoAbs against GM-CSF, or the three patients’ (P1-P3) plasma. NS: non-stimulated.

## Data Availability

All data are either included in the manuscript or available upon request.
