# Supplementary material for: Anti-GM-CSF Neutralizing Autoantibodies in Colombian Patients with Disseminated Cryptococcosis
Source: J Clin Immunol. 2023 Feb 23;43(5):921–32. doi: 10.1007/s10875-023-01451-5 (PMC9947894; doi:10.1007/s10875-023-01451-5)
Supplement: Supplementary file 1 — Supplementary file1 (PPTX 922 KB) Supplemental figure 1: Anti-GM-CSF auto-Ab titers in plasmas, diluted 1/50, from the three patients (P1-P3), healthy European individuals (n=35, white circles), patients previously shown to have anti-GM-CSF (n=5, gray diamond) or anti-IFN-γ (n=1, black and white diamond) auto-Abs, an APS-1 patient with auto-Abs against IL-17A, IL-17F, IL-22, IFN-α, and IFN-ω (n=1, black circle). Supplemental figure 2: Absence of neutralizing auto-Abs against type I IFNs in the plasma of the patients. Relative luciferase activity (RLA) ratio after stimulation with each of the IFN-α subtypes or IFN-ω at a concentration of 100 pg/mL, with a 1:10 dilution of plasma from seven healthy controls (negative control), an APS-1 patient (positive control), and the three patients with neutralizing anti-GM-CSF auto-Abs. Results are normalized against the RLA value obtained in the presence of plasma but absence of stimulation. Supplemental figure 3: Plasma from the three patients diluted 1:10 neutralizes up to 80 ng/mL GM-CSF. pSTAT5 levels assessed by flow cytometry on total PBMCs from a healthy donor stimulated with 5 ng/mL, 20 ng/mL, 40 ng/mL, or 80 ng/mL of GM-CSF or 100 ng/mL of IL-3, in the absence of plasma, or in the presence of a 1:10 dilution of plasma from two healthy controls (negative control), a patient with alveolar proteinosis PAP (positive control), or one of the three patients. NS: non-stimulated. Supplemental figure 4: STAT5 phosphorylation (p-STAT5), assessed by flow cytometry, upon stimulation with 5ng/mL rhGM-CSF (red) or 100ng/mL rhIL-3 (black) of control PBMCs in the presence of a 1:10, 1:100 or 1:1,000 dilution of plasma from two healthy individuals, three individuals previously described with neutralizing autoAbs against GM-CSF, or the three patients’ (P1-P3) plasma. NS: non-stimulated. [file 10875_2023_1451_MOESM1_ESM.pptx]

## Slide 1
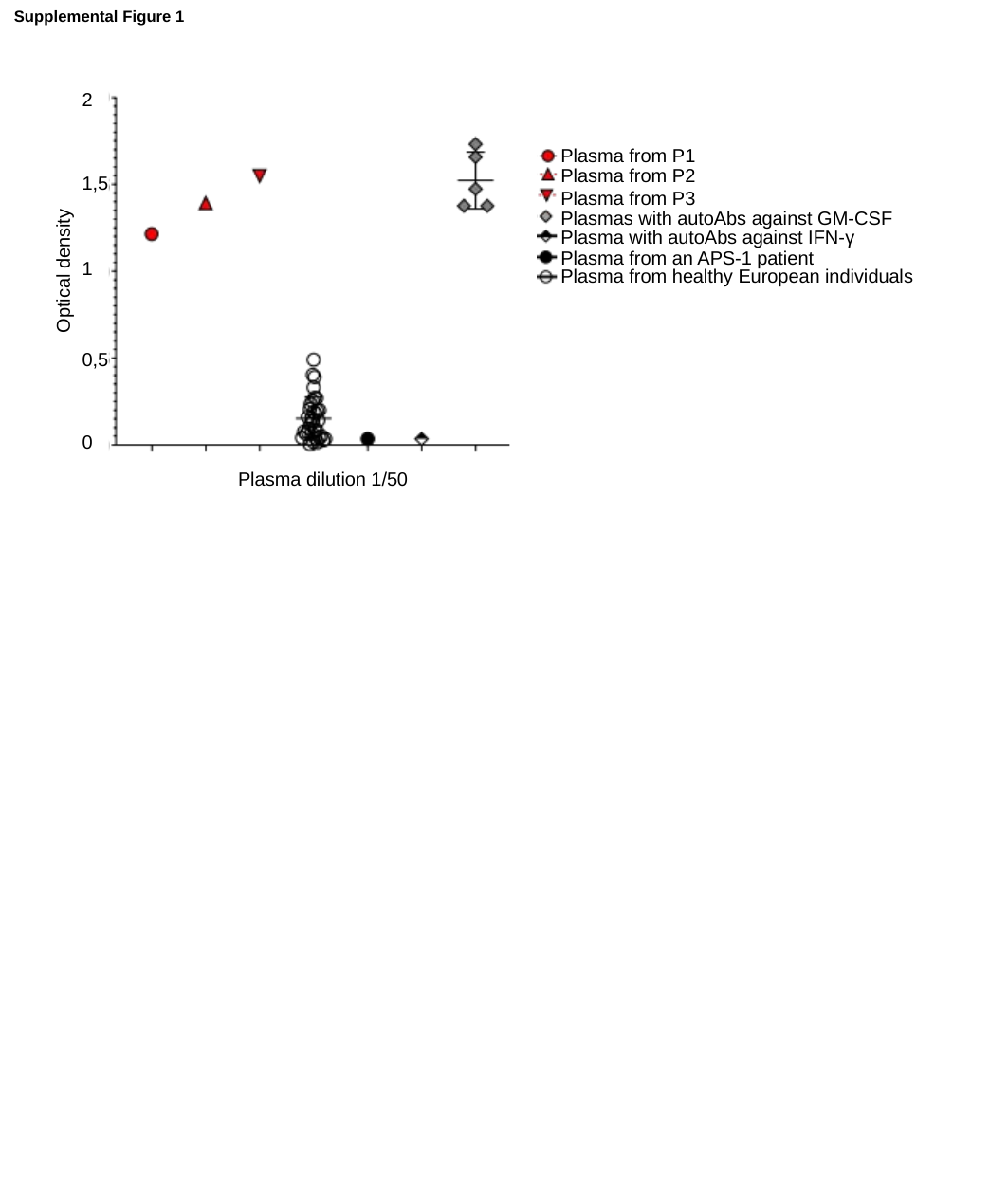

Supplemental Figure 1
2
Plasma from P1
Plasma from P2
1,5
Plasma from P3
Plasmas with autoAbs against GM-CSF
Plasma with autoAbs against IFN-γ
Plasma from an APS-1 patient
1
Optical density
Plasma from healthy European individuals
0,5
0
Plasma dilution 1/50

## Slide 2
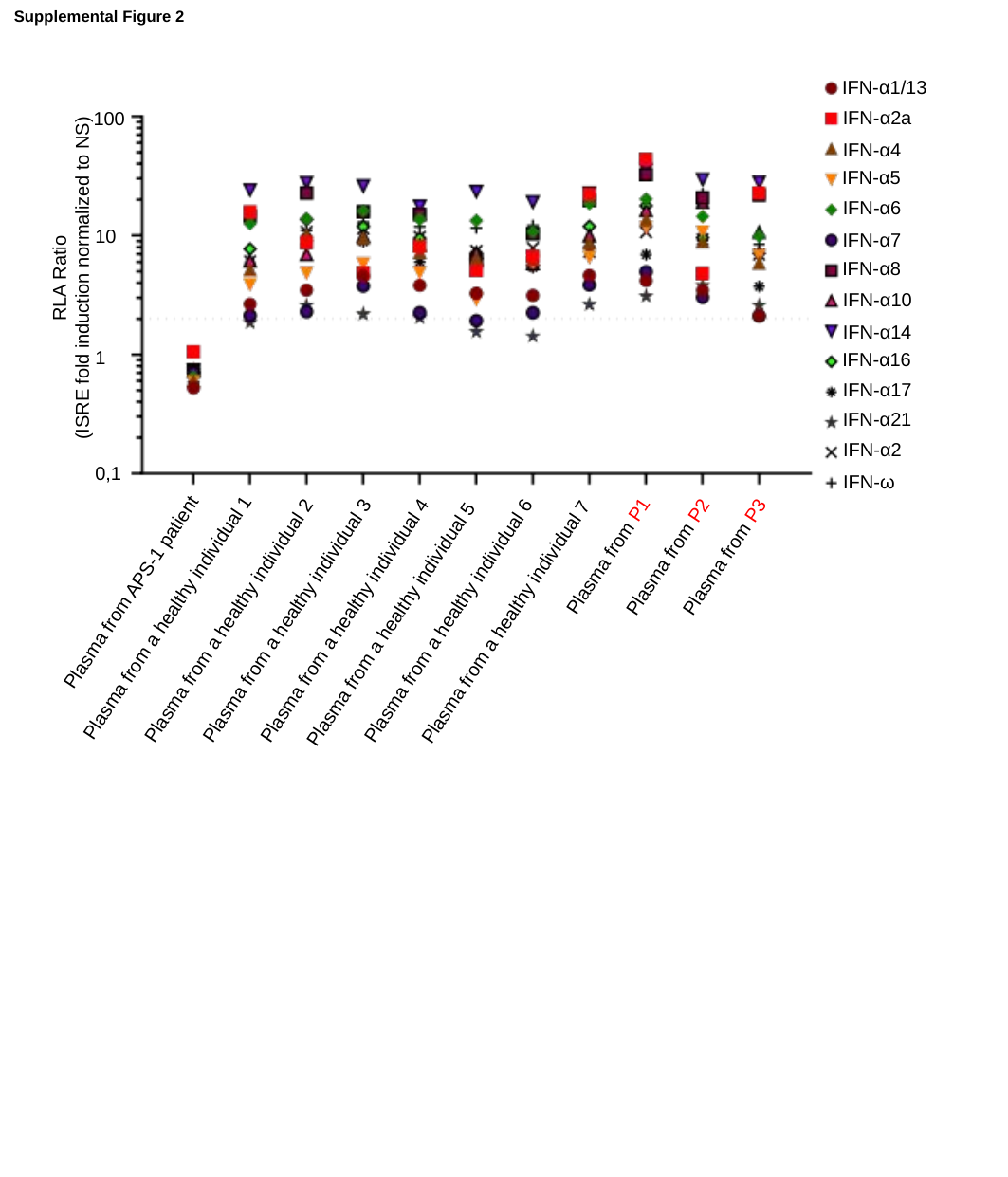

Supplemental Figure 2
IFN-α1/13
IFN-α2a
100
IFN-α4
IFN-α5
IFN-α6
10
IFN-α7
RLA Ratio
(ISRE fold induction normalized to NS)
IFN-α8
IFN-α10
IFN-α14
1
IFN-α16
IFN-α17
IFN-α21
IFN-α2
0,1
IFN-ω
Plasma from P1
Plasma from P2
Plasma from P3
Plasma from APS-1 patient
Plasma from a healthy individual 1
Plasma from a healthy individual 6
Plasma from a healthy individual 4
Plasma from a healthy individual 2
Plasma from a healthy individual 3
Plasma from a healthy individual 7
Plasma from a healthy individual 5

## Slide 3
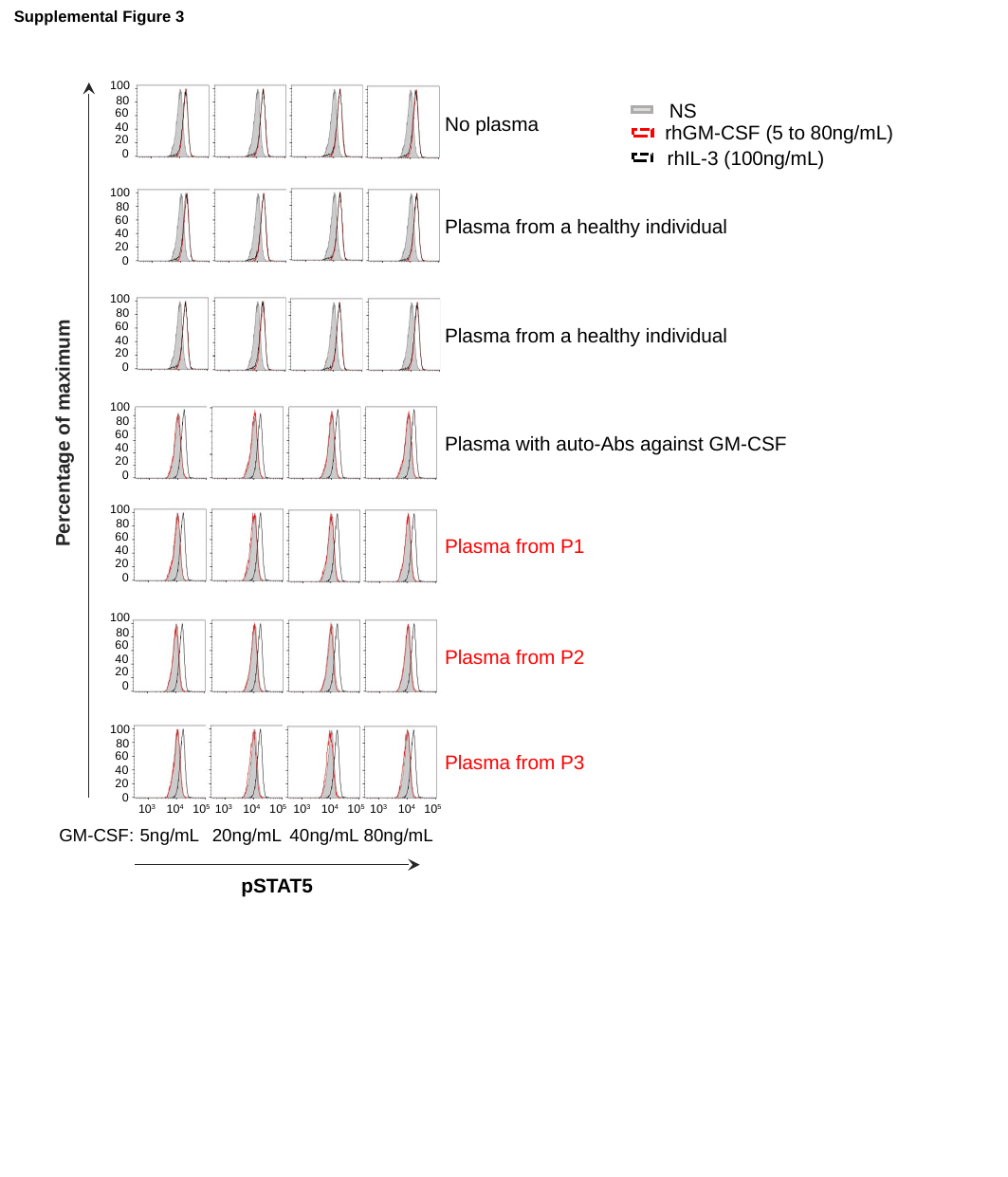

Supplemental Figure 3
100
80
60
40
20
0
NS
No plasma
rhGM-CSF (5 to 80ng/mL)
rhIL-3 (100ng/mL)
100
80
60
40
20
0
Plasma from a healthy individual
100
80
60
40
20
0
Plasma from a healthy individual
100
80
60
40
20
0
Percentage of maximum
Plasma with auto-Abs against GM-CSF
100
80
60
40
20
0
Plasma from P1
100
80
60
40
20
0
Plasma from P2
100
80
60
40
20
0
Plasma from P3
103
104
105
103
104
105
103
104
105
103
104
105
GM-CSF:
5ng/mL
20ng/mL
40ng/mL
80ng/mL
pSTAT5

## Slide 4
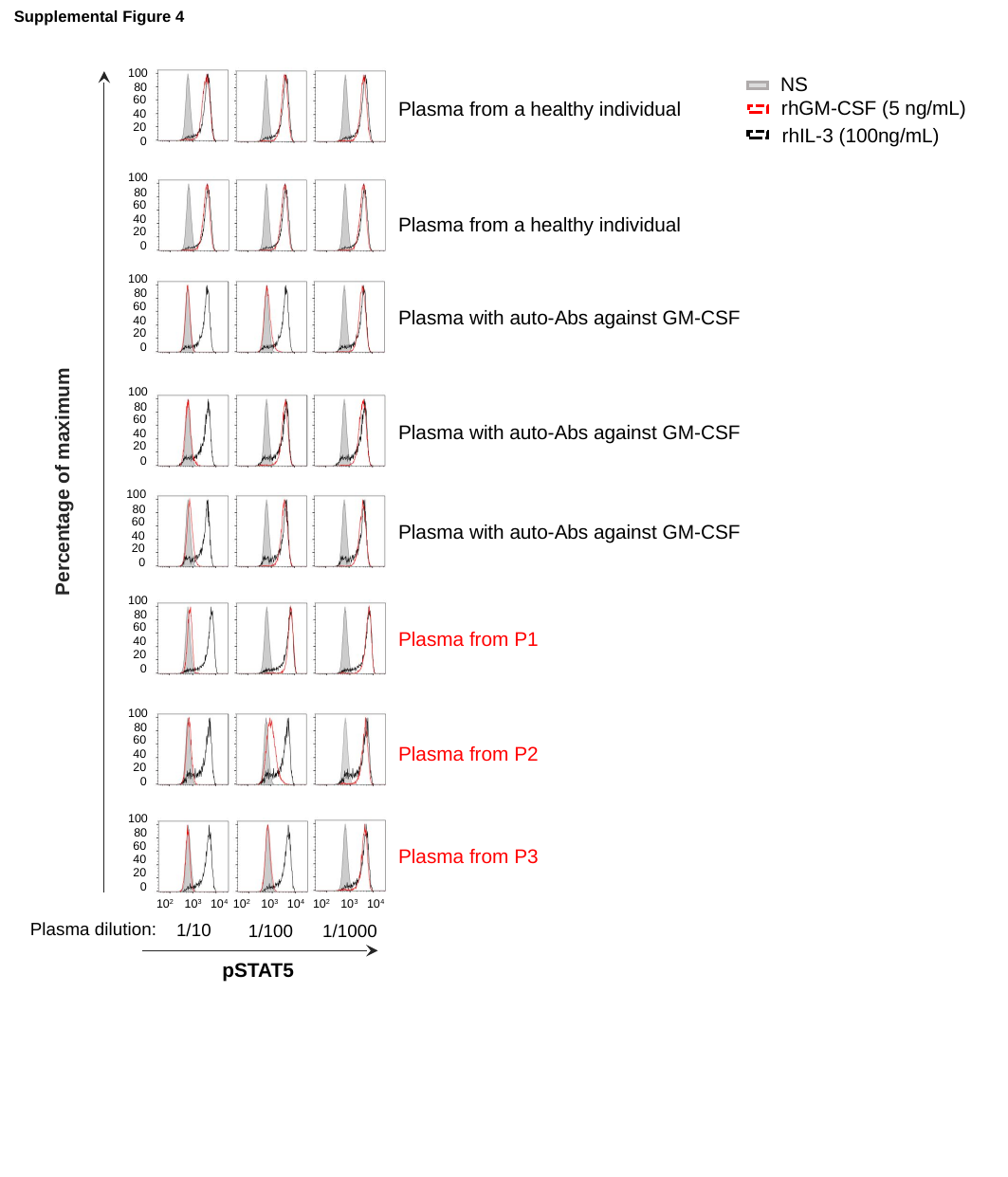

Supplemental Figure 4
100
80
60
40
20
0
NS
rhGM-CSF (5 ng/mL)
Plasma from a healthy individual
rhIL-3 (100ng/mL)
100
80
60
40
20
0
Plasma from a healthy individual
100
80
60
40
20
0
Plasma with auto-Abs against GM-CSF
100
80
60
40
20
0
Plasma with auto-Abs against GM-CSF
Percentage of maximum
100
80
60
40
20
0
Plasma with auto-Abs against GM-CSF
100
80
60
40
20
0
Plasma from P1
100
80
60
40
20
0
Plasma from P2
100
80
60
40
20
0
Plasma from P3
102
103
104
102
103
104
102
103
104
Plasma dilution:
1/10
1/100
1/1000
pSTAT5
